# Describe the outcomes of dysvascular partial foot amputation and how these compare to transtibial amputation: a systematic review protocol for the development of shared decision-making resources

**DOI:** 10.1186/s13643-015-0161-9

**Published:** 2015-12-04

**Authors:** Michael P Dillon, Stefania Fatone, Matthew Quigley

**Affiliations:** Discipline of Prosthetics and Orthotics, College of Science, Health and Engineering, La Trobe University, Melbourne, 3086 Australia; Northwestern University Prosthetic and Orthotic Centre, Feinberg School of Medicine, Northwestern University, 680 N Lake Shore Drive, Suite 1100, Chicago, IL 60611 USA

**Keywords:** Systematic review, Amputation, Partial foot, Transtibial, Shared decision-making, Epidemiology, Mortality, Mobility, Participation, Function, Quality of life

## Abstract

**Background:**

Helping people make well-informed decisions about dysvascular partial foot amputation is becoming increasingly important as improvements in diabetes care and vascular surgery make more distal amputations increasingly possible. The high rates of complications and reamputations associated with partial foot amputation are of concern, particularly given that transtibial amputation seems to result in similar outcomes (e.g., mobility and quality of life) with comparatively few complications and reamputations. The aim of this review is to describe the outcomes of dysvascular partial foot amputation and compare these to transtibial amputation. Results from the review are intended for use in the development of shared decision-making resources.

**Methods/design:**

A comprehensive range of databases—MEDLINE, EMBASE, PsycINFO, AMED, Cumulative Index of Nursing and Allied Health Literature (CINAHL), ProQuest Nursing and Allied Health, and Web of Science—will be searched using National Library of Medicine, Medical Subject Headings (MeSH) terms as well as title, abstract, and keywords relating to different amputation levels and outcomes of interest; specifically: incidence, prevalence, and rate of amputation; rate of mortality, wound failure, dehiscence, and time between index and ipsilateral reamputations; and mobility, functional ability, activity and participation, quality of life, pain, and psychosocial outcomes including depression and anxiety. Articles that meet the inclusion criteria will be hand-searched for relevant citations. A forward citation search using Google Scholar will be used to identify articles not yet indexed. Original research published in the English language after 1 January 2000 will be included. The McMaster Critical Review Forms will be used to assess methodological quality and identify sources of bias. Included articles will be independently appraised by two reviewers. Data will be extracted using a spreadsheet based on the Cochrane Consumers and Communication Review Group’s data extraction template by a primary reviewer and checked for accuracy and clarity by a second reviewer. Findings from the review will be reported as a narrative without meta-analysis given the anticipated heterogeneity of the literature.

**Discussion:**

Results from the review can be used in the design of shared decision-making resources to help inform difficult decisions about partial foot amputation.

**Systematic review registration:**

PROSPERO CRD42015029186

**Electronic supplementary material:**

The online version of this article (doi:10.1186/s13643-015-0161-9) contains supplementary material, which is available to authorized users.

## Background

Lower limb amputation is an all too common sequel to advanced peripheral vascular disease and the long-term effects of diabetes that results in a wide range of adverse health outcomes such as impaired mobility [1, 2], chronic pain [3], and depression [4]. These consequences of lower limb amputation often lead to significant disability and reduced quality of life [5, 6]. Therefore, it is not surprising that the decision to proceed with amputation surgery is a difficult one, even for people facing decisions about, so-called, *minor* or partial foot amputation.

Helping people make well-informed decisions about partial foot amputation has become increasingly important given that, over the last 15 years, there has been a dramatic shift in the types of lower limb amputation performed [7–11]. The incidence of transtibial amputation has declined steadily since about the year 2000 [7, 12–15], and there is some evidence that partial foot amputation has increased proportionately [7, 10, 13]. If these trends hold true into the future, the incidence of partial foot amputation is estimated to triple across the first half of this century [7].

However, there is some uncertainty about these observations given the way different health jurisdictions and researchers measure and report these data [16, 17]. For example, counting of first-ever (index) amputation underestimates the number of surgeries compared to counting all amputation procedures but may better capture the number of people affected [16, 17]. This is particularly problematic in people with partial foot amputation given that one third of amputations are revised to a higher level [18–22]. Many studies only count *major* amputations (e.g., above-the-ankle) [14, 23] and under-report the incidence of all lower limb amputations given that up to three quarters of all lower limb amputations are partial foot amputations [7, 24].

These variations in study design cloud our understanding of changes in types of lower limb amputations being performed; particularly the shift from transtibial to partial foot amputation. A better understanding of these changes is important to help plan for the increased number of people living with partial foot amputation and the specialist clinical services they require (e.g., wound care and high-risk foot clinics and prosthetic, orthotic, and pedorthic services).

The shift to a more distal, partial foot amputation will be seen by many as a significant improvement given the assumption that a more distal amputation results in better outcomes such as improved mobility [25], improved quality of life [26–28], and lower mortality [29–31]. However, partial foot amputation has been associated with a significant rate of failure and numerous complications. Between 30 and 50 % of people with partial foot amputation will experience complications including : dehiscence, ulceration, or complete failure of the wound to heal [22, 26, 27, 32–34]. Only about 50 % of all partial foot amputations heal with no appreciable difference based on the level of partial foot amputation [19–22, 35, 36]. The rate of healing of partial foot amputation is only about 10 % better in non-diabetic populations, making it difficult to conclude that the high failure rate is simply a reflection of advanced systemic disease [20–22]. About one third of people with an initial partial foot amputation will require revision surgery, irrespective of the level of partial foot amputation, even toe amputation [18–22, 36, 37]. The rates of failure and reamputation in people with partial foot amputation are disproportionately high when you consider that more than 80 % of all transtibial amputations heal and only about 10 % require subsequent amputation surgery on the same limb [36–39].

It is important to contextualize the high rates of complications and reamputations given that, at the point of amputation, people with dysvascular partial foot amputation have a very short life expectancy. About 25–40 % of people will die within 1 year of their dysvascular partial foot amputation, and the average life expectancy is less than 2 years [20, 36, 40]. The short life expectancy following dysvascular partial foot amputation makes it easier to appreciate just how important it is to help people make truly well-informed decisions about their healthcare.

This realization has led to renewed debate about the benefits and complications of partial foot amputation and how outcomes compare to transtibial amputation [41, 42]. Some authors have challenged the long-held belief that the high rates of complications and reamputations associated with partial foot amputation are worth the benefits, particularly given that key outcomes such as mobility [1, 2] and quality of life [43–46] are comparable in people with partial foot and transtibial amputation. However, concerns raised by other authors suggest that closer scrutiny of the evidence is necessary [47–49]. For example, much of the literature focuses on people with amputations through the midfoot (e.g., transmetatarsal amputation) and outcomes may be better for people with toe amputations [47, 48]. Similarly, some studies report very high rates of wound healing in people with partial foot amputation—comparable to those for people with transtibial amputation—but it is unclear what made some surgical and rehabilitation programs so much more effective [32, 50]. Research seems to have been focused on outcomes related to surgery or gait with little emphasis on participation or psychosocial outcomes. This is of particular concern given that depression and anxiety are common experiences. For example, people with partial foot amputation report being fearful and anxious about the prospect of future amputations, which does not seem to be the case for people with transtibial amputation [51].

A comprehensive systematic review on these topics is important to accurately describe the outcomes of partial foot amputation and how these compare to alternative surgical interventions, specifically transtibial amputation. Findings from this systematic review could support creation of shared decision-making resources [52] to help clinicians and patients make well-informed decisions about partial foot.

Shared decision-making is a consultative process whereby a clinician and patient jointly participate in making decisions about healthcare [52, 53]. It is recognized internationally as a hallmark of good clinical practice and a more effective means of influencing decisions about healthcare treatment compared to evidence summaries, best practice statements, clinical practice guidelines, or treatment algorithms [52]. The process of shared decision-making is seen as particularly valuable where there is uncertainty about the evidence for different treatment options or where decisions may be strongly influenced by an understanding of the potential benefits and harms or by a patient’s preferences and values [52]. As such, it may be particularly valuable given the uncertainties about the outcomes for dysvascular partial foot amputation and how these compare to alternative surgical options such as transtibial amputation.

Hence, the aims of this systematic review are to, first, describe the outcomes of dysvascular partial foot amputation with particular reference to the:

- Incidence, prevalence, or rate of amputation
- Rate of mortality, wound failure, dehiscence or skin breakdown (i.e., ulceration) as well as time between index and ipsilateral reamputations
- Mobility, function, participation, quality of life, pain (i.e., phantom or residual limb pain), and psychosocial outcomes (i.e., depression, anxiety, body image, self-esteem)

And second, compare the outcomes of partial foot and transtibial amputation with reference to the same outcomes.

## Methods/design

### Search strategy

A number of databases will be searched individually using the OVID platform: MEDLINE, EMBASE, PsycINFO, and AMED. Stand-alone searches will also be conducted using : Cumulative Index of Nursing and Allied Health Literature (CINAHL), ProQuest Nursing and Allied Health, and Web of Science.

Where possible, these databases will be searched using the National Library of Medicine, Medical Subject Headings (MeSH) *amputation* as well as *amputee*. These MeSH terms are consistent with the root (e.g., amputation) and hierarchical branches (e.g., amputation/statistics and numerical data) commonly used to index research involving people with lower limb amputation. We will not employ MeSH terms related to the outcome measures of interest given that articles are not reliably indexed using these terms. For example, many studies that are indexed using the MeSH term *quality of life* do not actually measure that outcome. By contrast, studies that actually measure quality of life, but use outcome measures such as the Sickness Impact Profile, are often not indexed using the MeSH term *quality of life*. MeSH terms will be “exploded” to capture all branches of the MeSH hierarchy given the small additional increase in the search yield compared to a “focused” search using relevant hierarchical branches of the MeSH tree.

A list of search terms related to the population (e.g., amputation level) and outcome measures (e.g., quality of life), as well as their synonyms and acronyms, will be used in conjunction with wildcards and Boolean operators as part of a title, abstract, and keyword search. Given that some databases do not provide these exact field codes, alternatives will be used as necessary. For example, ProQuest Nursing and Allied Health does not include an author keyword field and as such, the alternative field code, identifiers (IF), will be used.

Each search strategy will be rigorously developed and tested. Individual search terms relating to each topic will be identified based on reading relevant systematic reviews, original research articles as well as searching databases of outcomes measures (e.g., rehabmeasures.org). Individual search terms will be constructed, tested, and adapted to ensure that variations in spelling, punctuation, acronyms, as well as adaptations to the names of outcomes (e.g., Prosthesis Evaluation Questionnaire is sometimes incorrectly described as the Prosthesis Experience Questionnaire) are captured using wildcards and proximity operators. Generic acronyms will be removed from the search to improve the relevance of the results (e.g., the acronym for the *Keele Assessment of Participation* (KPA) will likely identify articles measuring pressure in kiloPascals, kPa). These search terms will be built into larger search strings that include parentheses, quotation marks, and Boolean operators to control the logic of the search as appropriate to each database. These search strings will be tested by comparing the search terms to the words highlighted in the title, abstract, keywords, and MeSH terms included in the results. Errors will be queried, and the search strings will be adapted and retested as necessary. The precision and comprehensiveness of each search strategy will be evaluated by comparing the search results to a bank of known articles on each topic. Articles included in the bank will cover a range of journal titles and publication years. When the search fails to identify known articles included in the bank, the search strategy will be queried, adapted, and retested.

Given that the authors are native English speakers and that restriction of non-English language articles does not seem to alter the outcome of systematic reviews and meta-analyses [54, 55], all searchers will be limited to the English language. Search strategies will also be limited by time (i.e., 1 January 2000 to end search date) given that, in the decade prior to the year 2000, lower limb amputation above the ankle was increasingly common and the relative risk of amputation was significantly higher than has been the case since [12, 14, 15]. Treatments for diabetic complications and amputation surgery have also changed markedly over time with the commonplace use of revascularization surgery prior to amputation as an illustrative example [15]. Similarly, advances in prosthetic technology have had a dramatic effect on the interventions provided for people with partial foot and transtibial amputation (e.g., carbon fiber ankle foot orthoses and silicone liners), and there is some evidence of their effect on the outcomes of interest [56, 57]. As such, it is felt that literature prior to the year 2000 will not provide the most current evidence with which to inform answers to our research aims or subsequent shared decision-making resources. Some databases will be further limited to the type of article to improve precision of the yield. For example, ProQuest Nursing and Allied Health will be limited to “peer reviewed articles” given that the database also indexes magazine and trade journals among other literature.

In keeping with the Preferred Reporting Items for Systematic Reviews and Meta-Analyses (PRISMA) guidelines [58], an illustrative search is presented for one database (Table 1). Similarly, detailed searchers will be constructed for each outcome and database included in the review.

**Table 1:** Example search for the CINAHL database to identify quality of life literature for people with dysvascular partial foot and transtibial amputation

Search	Field code	Search term(s)
1.	MH	“Amputation”
2.	MH	“Amputees”
3.	TI, AB, SU	(amput* AND (major OR lowerlimb* OR “lower limb”* OR “lower extremit*” OR “limb loss” OR LEA OR LLA))
4.	TI, AB, SU	(amput* AND (transtibial OR “trans tibial” OR belowknee OR “below knee” OR (below W2 knee) OR TTA OR BKA))
5.	TI, AB, SU	(amput* AND (minor OR “partial foot” OR Chopart* OR Lisfranc* OR tarsometatarsal OR transmetatarsal OR midtarsal OR “mid tarsal” OR midfoot OR “mid foot” OR ray OR phalangeal OR metatarsophalangeal OR toe* OR transtarsal OR “trans tarsal” OR TMT OR TMA OR MTP OR PFA))
6.		1 OR 2 OR 3 OR 4 OR 5
7.	TI, AB, SU	SF 36 OR SF36 OR “Medical Outcome* Study Short Form*” OR “Medical Outcome* Study Short-Form*” OR “MOS SF 36” OR “MOS SF36” OR “Sickness Impact Profile*” OR “SIP” ((“Trinity Amputation and Prosthe* Experience”) W1 (Survey OR Scale*)) OR TAPES OR “Prosthe* Evaluation Questionnaire” OR PEQ OR “WHO QOL BREF” OR “WHO QOLBREF” OR “WHOQOLBREF” OR ((WHO OR “World Health Organi#ation”) W1 (“Quality of Life BREF” OR “Quality of Life Scale”)) OR “RAND36” OR “RAND 36” OR “Orthotic*and Prosthetic* User* Survey” OR OPUS OR ((“Health Related”) W1 “Quality of Life”) OR HRQOL OR “Life Satisfaction Questionnaire* 9” OR “LiSat 9” OR “Satisfaction With Life Scale” OR SWLS OR “Quality of Well Being” OR QWB* OR “Quality of Life Index” OR QLI OR “EuroQOL*” OR “Euro QOL” OR EQ5D OR “EQ 5D” OR “Assessment of Quality of Life” OR AQoL OR (Orthotic* W2 “prosthetic* user* survey”) OR “Attitude to Artificial Limb* Questionnaire” OR AALQ
8		6 AND 7
9.		Limit 8 to English language
10.		Limit 9 to publication date: 01.01.2000 to 31.12.2015
11.		Limit 10 to peer reviewed, academic journals

Field codes: *MH* exact major and minor subject headings (MeSH, National Library of Medicine Medical Subject Headings), *TI* title, *AB* abstract, *SU* subject

Reference lists of articles that meet the inclusion criteria will be hand-searched to ensure that relevant publications have not been overlooked.

Given the significant lag between publication of early online articles and indexation in databases such as MEDLINE, we will conduct a forward citation search using articles that meet the inclusion criteria. We will perform the forward citation search using Google Scholar given that the proprietary algorithm does not rely on indexation in any database. The use of Google Scholar to search the literature not indexed elsewhere is an increasingly accepted practice to augment traditional database searches [59–61].

### Data management

Results from each database search will be exported directly into a shared online library in EndNote X7.2.1 (Thomson Reuters Inc.). Using the “find duplicates” feature in EndNote, duplicate records will be checked and deleted. Hand-searching the EndNote records for duplicates will also be undertaken given that small differences in the bibliographic information (e.g., inclusion of a middle initial in the authors’ name) often means that duplicates will not be automatically detected. Full-text copies of articles will be sourced using the “find full text” feature in EndNote or manually retrieved (e.g., through interlibrary loan services). Where articles are manually retrieved, electronic versions will be subsequently linked to the relevant EndNote record.

EndNote records will then be exported into a Microsoft Excel spreadsheet using a custom-made EndNote output style. The EndNote output style will allow key bibliographic information about each reference (i.e., EndNote reference number, authors, title, year, journal title, volume, issue, page, and abstract) to be exported in a tab delimited format such that it can be opened in Excel. The resulting Excel spreadsheet will include each article on a single row and separate columns for the bibliographic information. Additional columns will be added to allow decisions about inclusion/exclusion to be recorded, reasons for exclusion, and to record information about the number of full-text articles inspected. The same spreadsheet will be expanded for data extraction and to record details of the critical appraisal of each article as detailed in later sections of the protocol.

To record details necessary for completion of the PRISMA flowchart, a separate tab in the Excel spreadsheet will be used to record details about the number of articles retrieved from each database, duplicates removed, records screened based on title and abstract or full-text, as well as the number of additional records retrieved from hand-searching and forward citation searches.

### Selection process

The following criteria will be used to determine inclusion:

1. Peer reviewed studies of original research
2. Studies published in the English language
3. Studies published since 1 January 2000
4. For the first aim—describe outcomes of dysvascular partial foot amputation—studies must include discrete cohort(s) with dysvascular partial foot amputation (with or without diabetes), acknowledging that some studies will include subgroups with different levels of partial foot amputation
5. For the second aim—compare outcomes of dysvascular partial foot and transtibial amputation—studies must include discrete cohorts with dysvascular partial foot amputation and transtibial amputation (with or without diabetes).
6. Studies that measure the outcome(s) of interest, specifically:
  - Incidence, prevalence, or rate of amputation
  - Rate of mortality, wound failure, dehiscence or skin breakdown (i.e., ulceration) as well as time between index and ipsilateral reamputations
  - Mobility, functional ability, participation, quality of life, pain, or psychosocial outcomes

It is anticipated that the operational definitions and time points of these outcomes will vary across studies. As such, studies that meet these criteria will be included irrespective of how the outcome has been defined or the time point at which it was measured. By way of example, studies reporting the rate of mortality will be included irrespective of the time point at which the outcome was measured (e.g., 1, 3, or 5 years post amputation). Given that some of the outcomes are not so readily defined, we include the following definitions. For the purposes of this review, mobility is defined as the ability to move independently from one place to another [62, 63]. By virtue of this definition, outcomes focusing on distance and time, or those that decompose movement into discrete subtasks (e.g., sit to stand), will be included along with community measures of mobility that reflect the potential impairment that results from environmental barriers common to many day-to-day mobility tasks in the community. Participation is defined as involvement in all areas of life [64] and thereby includes outcomes that capture involvement in a broad range of life situations such as family roles, self-care needs, social activities, work, and education. Functional ability is defined as the skill to perform activities [63]—typically activities of daily living, such as self-care—and the degree to which difficulty limits independence with these activities. Psychological outcomes of interest include depression, anxiety, body image, and self-esteem given publications highlighting these experiences among people with partial foot amputation [51, 65]. Similarly, measures of phantom and residual limb pain will also be recorded.

By virtue of the inclusion criteria, studies with a heterogeneous sample of different amputation levels (e.g., all above-the-ankle amputations) or causes (e.g., trauma and peripheral vascular disease) will be excluded. While editorials, letters, conference abstracts, and opinion pieces will be excluded, there will be no other restrictions regarding study design.

Definitions of transtibial and partial foot amputation will be consistent with the International Standards Organization (ISO) definitions [66] and as such all levels of partial foot amputation, including toe amputation, will be included. By virtue of the ISO definition, ankle disarticulation (i.e., Syme amputation) is not considered a partial foot amputation and as such, studies focused on this level of amputation will be excluded.

Search results will be screened by one investigator based on review of the title and abstract. In cases where insufficient detail is reported in the abstract, the full-text article will be retrieved. Given that the inclusion criteria are unambiguous and do not require complex judgment, it will be unnecessary to routinely involve two investigators in the screening process [67]. On occasion, a second opinion may be sought from another investigator and any disagreement will be resolved through discussion until consensus. Following screening based on title and abstract, full-text articles will be retrieved and independently reviewed by two investigators to confirm inclusion.

### Quality appraisal/risk of bias in individual studies

We will use the McMaster Critical Review Forms [68, 69] to assess methodological quality and identify sources of bias in included articles. The McMaster Critical Review Forms are one of the few appraisal tools appropriate for use with a wide variety of study designs [70] and include structured guidelines to reduce the likelihood of errors with use [71]. The McMaster Critical Review Forms meet acceptable standards for content and initial construct validity as well as inter-rater and test re-test reliability [72]. Results from the quality appraisal will be reported in tabular format using Microsoft Excel and include detailed comments to support the checklist items, as illustrated in Table 2.

**Table 2:** Example table showing results of a quality appraisal for two quality of life (QoL) studies using the McMaster Critical Review Form for quantitative studies [45]

	Aim + background		Study design	Sample			Outcomes		Intervention			Results				Conclusion
Study	A	B	C	D	E	F	G	H	I	J	K	L	M	N	O	P
Boutoille et al. [46]			Case-control	34			Y	Y				Y				
				25-9^a^												
	Reviewer comments: Unclear description of aim and background. New results were presented in the discussion. QoL in the partial foot and transtibial amputee cohorts were compared to non-amputee cohort with foot ulceration but not to each other. Detailed sample demographic information was not provided. Co-intervention: the foot ulcer group was receiving active treatment while the amputee group had completed rehabilitation. Power bias present in assessment of capacity for inclusion, as the participants’ doctor decided if they were able to complete the survey. High risk of type-1 error due to multiple *t* tests employed. Large variability in the results, making it difficult to detect meaningful differences.															
Peters et al. [43]	Y	Y	Case-control	124	Y		Y	Y				Y		Y		
				35-89^a^												
	Reviewer comments: Control bias: the case and control groups were significantly different in terms of: gender, duration of diabetes, and degree of neuropathy. QoL assessed with Sickness Impact Profile, focusing on functional status. Data pooled for persons with transtibial and transfemoral amputations (high level) and all levels of partial foot amputation (mid-level). A large variability in the results makes it difficult to detect meaningful differences. High chance of a Type 1 error given between group comparisons of multiple dependent variables.															

Quality assessment items from McMaster University Critical Review Form—quantitative studies are listed as per the following key: A: Was the study purpose stated clearly?; B: Was relevant background literature reviewed?; C: Study design; D: Sample size (n) and the number of cases versus controls as applicable; E: Was the sample described in detail?; F: Was sample size justified?; G: Were the outcome measures reliable?; H: Were the outcome measures valid?; I: Was intervention described in detail?; J: Was contamination avoided?; K: Was co-intervention avoided?; L: Were results reported in terms of statistical significance?; M: Were the analysis method(s) appropriate?; N: Was clinical importance reported?; O: Were dropouts reported?; P: Were conclusions appropriate given study methods and results? Note: For clarity, only questions with affirmative responses have been shown

^a^Number of cases versus controls

### Data extraction

A data extraction spreadsheet will be developed in Microsoft Excel based on the Cochrane Consumers and Communication Review Group’s data extraction template to allow socio-demographic (e.g., age, sex, etiology, level of amputation, comorbidities), methodological (e.g., aim, study design, recruitment method, inclusion criteria), results (e.g., outcome measures), and quality appraisal details (using checklist items and comments) to be systematically recorded [73]. The layout of the spreadsheet and the included fields will be based on previous systematic reviews that included people with partial foot or transtibial amputation, similar outcome measures, and quality appraisal tools [45, 57, 74]. In keeping with recommendations to reduce error rates and omissions and improve reliability of data extraction, column headings will include intuitive acronyms, units of measurement, and fields for comments. For example, comments embedded in the column headings for the critical appraisal checklist items will include the actual question and decision rules so that reviewers can “scroll-over” the column heading for these details. Where possible, pull-down menus will be used to standardize data entry (e.g., list of study designs) and coding (e.g., yes/no/unclear/not reported/not applicable).

Prior to implementation, the data extraction spreadsheet will be piloted and refined. A number of articles covering the range of topics will be appraised by each of the reviewers and data extracted. Adaptations to the spreadsheet will be made in response to feedback on the pilot.

Included articles will be independently appraised by two reviewers with good knowledge of the content area and experience in conducting systematic reviews, including the process of data extraction and verification. Data will be extracted by a primary reviewer and checked for accuracy and clarity by a second reviewer. Given the scope of the review, single data extraction with independent verification is preferred to duplicate data extraction given the significant time saving (average 50 min per article) [75] and that both approaches result in very similar error rates (single extraction 17 %; double extraction 14.5 %) [75] that have been shown to be inconsequential to the final outcome [75, 76]. Disagreements or inconsistencies in the data extraction or critical appraisal will be resolved through discussion until consensus. As necessary, a third reviewer will be called upon to also appraise the article and contribute to the consensus decision. Authors of the original research will be contacted for additional information or to clarify aspects of the method design as deemed necessary.

Where data for the same participants has been reported across multiple studies, subject numbers, demographics, and outcomes will be compared for discrepancies. If there is uncertainty about the similarity of the study participants and results, authors of the original research will be contacted for clarification. Where data for the same subjects is reported across multiple studies, reference will be made to all the studies but data will be treated as a single source.

### Data summary and reporting

Findings from the review will be reported as a narrative without meta-analysis given that previous reviews involving people with partial foot amputation [42, 45, 57] suggest that there will be few studies on each outcome and these will be heterogeneous in terms of study design, outcome measures used, and subjects included, thus making meta-analysis inappropriate. As an illustrative example, a recent quality of life review [45] found just two case-controlled studies reporting data in people with partial foot amputation and transtibial amputation (Table 2). Each study used a different outcome measure, and details about the participants’ sex or the presence of comorbidities were not adequately reported, which made it difficult to assess the degree to which participants were similar across these studies (Table 2). This illustrative example typifies much of the literature involving people with limb loss and highlights why narrative reviews have been widely used [45, 57, 77].

In this review, the result narratives will be presented by topic (e.g., mobility) and the literature characterized in terms of study designs, subject characteristics, and outcome measures used. Studies will also be characterized in terms of their quality/risk of bias using the findings of the critical appraisal. Common issues with internal and external validity will be discussed with a specific focus on limitations that lead to imprecision, indirectness, inconsistency, and publication bias [78]. The extent to which these issues impact the results will be discussed and lead to an understanding about which studies engender the most confidence in the results and why. Where possible, results will be reported with a breakdown by level of partial foot amputation and comparisons to the outcomes of people with transtibial amputation will be made.

## Discussion

### Evidence for shared decision-making resources

We hope that by critically apprising and synthesizing evidence on the outcomes of partial foot amputation, we will be able to include information in future shared decision-making resources that is consistent with a well-informed interpretation of the current research evidence. In this way, the shared decision-making resources we create can help people engage in meaningful and often difficult discussions about dysvascular partial foot amputation given uncertainties about the outcomes and how these compare to alternative surgical options such as transtibial amputation.

### Method design considerations

There are a number of considerations that influence the design of the protocol for this systematic review that are important to acknowledge. The protocol was deliberately designed to address the primary aim of the review, that is, to describe the outcomes of dysvascular partial foot amputation. While the secondary aim is to compare outcomes between people with dysvascular partial foot and transtibial amputation, it is important to realize that it is largely cohort studies, not randomized control trials, that will provide the most appropriate evidence to inform this aim, as it would be unethical to randomize people to receive either a partial foot or transtibial amputation given that such decisions are highly influenced by a patient’s preferences and values [52]. It should come as no surprise that we do not expect any randomized control trials will be included in this review. By logical extension, the risk of bias in individual studies as well as confidence in the cumulative evidence cannot be judged by criteria suited to randomized control trials. As such, we hope readers appreciate our approach to assessing the risk of bias as well as describing the strength of the body of the evidence using a narrative approach.

## Additional files

Additional file 1:
**PRISMA-P 2015 checklist—recommended items to include in a systematic review protocol.**
Additional file 2:
**Grant award letter from the funding body.**

## Abbreviations

MeSH: National Library of Medicine, Medical Subject Headings
PRISMA: Preferred Reporting Items for Systematic Reviews and Meta-Analyses
ISO: International Standards Organization

## References

[1] Czerniecki JM, Turner AP, Williams RM, Hakimi KN, Norvell DC (2012). Mobility changes in individuals with dysvascular amputation from the presurgical period to 12 months postamputation. Arch Phys Med Rehabil.

[2] Norvell DC, Turner AP, Williams RM, Hakimi KN, Czerniecki JM (2011). Defining successful mobility after lower extremity amputation for complications of peripheral vascular disease and diabetes. J Vasc Surg.

[3] Ehde DM, Czerniecki JM, Smith DG, Campbell KM, Edwards WT, Jenson MP (2000). Chronic phantom sensations, phantom pain, residual limb pain, and other regional pain after lower limb amputation. Arch Phys Med Rehabil.

[4] Behel JM, Rybarczyk B, Elliott TR, Nicholas JJ, Nyenhuis D (2002). The role of perceived vulnerability in adjustment to lower extremity amputation: a preliminary investigation. Rehabil Psychol.

[5] Sinha R, van den Heuvel WJA, Arokiasamy P (2011). Factors affecting quality of life in lower limb amputees. Prosthet Orthot Int.

[6] Sinha R, van den Heuvel WJ, Arokiasamy P, van Dijk JP (2014). Influence of adjustments to amputation and artificial limb on quality of life in patients following lower limb amputation. Int J Rehabil Res.

[7] Dillon MP, Kohler F, Peeva V (2014). Incidence of lower limb amputation in Australian hospitals from 2000 to 2010. Prosthet Orthot Int.

[8] Trautner C, Haastert B, Mauckner P, Gatcke LM, Giani G (2007). Reduced incidence of lower-limb amputations in the diabetic population of a German city, 1990-2005: results of the Leverkusen Amputation Reduction Study (LARS). Diabetes Care.

[9] van Houtum WH, Rauwerda JA, Ruwaard D, Schaper NC, Bakker K (2004). Reduction in diabetes-related lower-extremity amputations in The Netherlands: 1991-2000. Diabetes Care.

[10] Vamos EP, Bottle A, Edmonds ME, Valabhji J, Majeed A, Millett C (2010). Changes in the incidence of lower extremity amputations in individuals with and without diabetes in England between 2004 and 2008. Diabetes Care.

[11] Fortington LV, Rommers GM, Postema K, van Netten JJ, Geertzen JH, Dijkstra PU (2013). Lower limb amputation in Northern Netherlands: unchanged incidence from 1991-1992 to 2003-2004. Prosthet Orthot Int.

[12] Li Y, Burrows NR, Gregg EW, Albright A, Geiss LS (2012). Declining rates of hospitalization for nontraumatic lower-extremity amputation in the diabetic population aged 40 years or older: U.S., 1988-2008. Diabetes Care.

[13] Lombardo FL, Maggini M, De Bellis A, Seghieri G, Anichini R (2014). Lower extremity amputations in persons with and without diabetes in Italy: 2001-2010. PLoS One.

[14] Ikonen TS, Sund R, Venermo M, Winell K (2010). Fewer major amputations among individuals with diabetes in Finland in 1997-2007: a population-based study. Diabetes Care.

[15] Gregg EW, Li Y, Wang J, Burrows NR, Ali MK, Rolka D (2014). Changes in diabetes-related complications in the United States, 1990-2010. N Engl J Med.

[16] Jeffcoate WJ (2005). The incidence of amputation in diabetes. Acta Chir Belg.

[17] Jeffcoate WJ, van Houtum WH (2004). Amputation as a marker of the quality of foot care in diabetes. Diabetologia.

[18] Izumi Y, Satterfield K, Lee S, Harkless LB (2006). Risk of reamputation in diabetic patients stratified by limb and level of amputation: a 10-year observation. Diabetes Care.

[19] Elsharawy MA (2011). Outcome of midfoot amputations in diabetic gangrene. Ann Vasc Surg.

[20] Landry G, Silverman D, Liem T, Mitchell E, Moneta G (2011). Predictors of healing and functional outcome following transmetatarsal amputations. Arch Surg.

[21] Nguyen TH, Gordon IL, Whalen D, Wilson SE (2006). Transmetatarsal amputation: predictors of healing. Am Surg.

[22] Pollard J, Hamilton GA, Rush SM, Ford LA (2006). Mortality and morbidity after transmetatarsal amputation: retrospective review of 101 cases. J Foot Ankle Surg.

[23] Eskelinen E, Lepantalo M, Hietala EM, Sell H, Kauppila L, Maenpaa I (2004). Lower limb amputations in Southern Finland in 2000 and trends up to 2001. Eur J Vasc Endovasc Surg.

[24] Driver VR, Madsen J, Goodman RA (2005). Reducing amputation rates in patients with diabetes at a military medical center: the limb preservation service model. Diabetes Care.

[25] Larsson J, Agardh C, Apelqvist J, Stenstrom A (1998). Long-term prognosis after healed amputation in patients with diabetes. Clin Orthop Relat Res.

[26] Imam U, Elsawy A, Balbaa A (2007). Functional outcome and complications of partial foot amputations in diabetics. Egypt J Surg.

[27] Dudkiewicz I, Schwarz O, Heim M, Herman A, Siev-New I (2001). Trans-metatarsal amputation in patients with diabetic foot: reviewing 10 years experience. Foot.

[28] Goktepe AS, Cakir B, Yilmaz B, Yazicioglu K (2010). Energy expenditure of walking with prostheses: comparison of three amputation levels. Prosthet Orthot Int.

[29] Attinger CE, Brown BJ (2012). Amputation and ambulation in diabetic patients: function is the goal. Diabetes Metab Res Rev.

[30] Brown M, Tang W, Patel A, Baumhauer J (2012). Partial foot amputation in patients with diabetic foot ulcers. Foot Ankle Int.

[31] Evans KK, Attinger CE, Al-Attar A, Salgado C, Chu CK, Mardini S (2011). The importance of limb preservation in the diabetic population. J Diabetes Complications.

[32] Sage R, Pinzur MS, Cronin R, Preuss HF, Osterman H (1989). Complications following midfoot amputation in neuropathic and dysvascular feet. J Am Podiatr Med Assoc.

[33] Santi MD, Thoma BJ, Chambers RB (1993). Survivorship of healed partial foot amputations in dysvascular patients. Clin Orthop.

[34] Mueller M, Allen B, Sinacore D (1995). Incidence of skin breakdown and higher amputation after transmetatarsal amputation: implications for rehabilitation. Arch Phys Med Rehabil.

[35] Stone PA, Back MR, Armstrong PA, Flaherty SK, Keeling WB, Johnson BL (2005). Midfoot amputations expand limb salvage rates for diabetic foot infections. Ann Vasc Surg.

[36] Thomas SR, Perkins JM, Magee TR, Galland RB (2001). Transmetatarsal amputation: an 8-year experience. [see comment]. Ann R Coll Surg Engl.

[37] Dillingham TR, Pezzin LE, Shore AD (2005). Reamputation, mortality, and health care costs among persons with dysvascular lower-limb amputations. Arch Phys Med Rehabil.

[38] Barber GG, McPhail NV, Scobie TK, Brennan MC, Ellis CC (1983). A prospective study of lower limb amputations. Can J Surg.

[39] Jordan RW, Marks A, Higman D (2012). The cost of major lower limb amputation: a 12-year experience. Prosthet Orthot Int.

[40] Kristensen MT, Holm G, Kirketerp-Moller K, Krasheninnikoff M, Gebuhr P (2012). Very low survival rates after non-traumatic lower limb amputation in a consecutive series: what to do?. Interact Cardiovasc Thorac Surg.

[41] Dillon M, Fatone S, Morris M (2014). Partial foot amputation may not always be worth the risk of complications. Med J Aust.

[42] Dillon M, Fatone S (2013). Deliberations about the functional benefits and complications of partial foot amputation: do we pay heed to the purported benefits at the expense of minimizing complications?. Arch Phys Med Rehabil.

[43] Peters EJ, Childs MR, Wunderlich RP, Harkless LB, Armstrong DG, Lavery LA (2001). Functional status of persons with diabetes-related lower-extremity amputations. Diabetes Care.

[44] Quigley M, Dillon MP, Duke E. Comparison of quality of life in people with partial foot and transtibial amputation: a pilot study. Prosthet Orthot Int. 2015. epub ahead of print. doi:10.1177/0309364614568414 10.1177/0309364614568414 25716956

[45] Quigley MJ, Dillon MP. Quality of life in persons with partial foot and transtibial amputation: a systematic review. Prosthet Orthot Int. 2014. epub ahead of print. doi: 10.1177/0309364614546526. 10.1177/0309364614546526 25185154

[46] Boutoille D, Féraille A, Maulaz D, Krempf M (2008). Quality of life with diabetes-associated foot complications: comparison between lower-limb amputation and chronic foot ulceration. Foot Ankle Int.

[47] Lazzarini PA, Malone M, Wraight P (2014). Letter RE: Dillon et al. (2014) Partial foot amputations may not always be worth the risk of complications. Med J Aust.

[48] Norman PE, Schoen DE, Nedkoff LJ (2014). Letter RE: Dillon et al. (2014) Partial foot amputations may not always be worth the risk of complications. Med J Aust.

[49] Dillon MP, Fatone S, Morris ME (2014). Letter RE: Dillon et al. (2014) Partial foot amputations may not always be worth the risk of complications. Med J Aust.

[50] Pinzur MS, Kaminsky M, Sage R, Cronin R, Osterman H (1986). Amputations at the middle level of the foot. J Bone Joint Surg Am.

[51] Livingstone W, Mortel TF, Taylor B (2011). A path of perpetual resilience: exploring the experience of a diabetes-related amputation through grounded theory. Contemp Nurse.

[52] Hoffman TC, France L, Simmons MB, McNamara K, McCaffery K, Travena LJ (2014). Shared decision making: what do clinicians need to know and why should they bother?. Med J Aust.

[53] Elwyn G, Frosch D, Thomson R, Joseph-Williams N, Lloyd A, Kinnersley P (2012). Shared decision making: a model for clinical practice. J Gen Intern Med.

[54] Moher D, Pham B, Lawson M, Klassen T (2003). The inclusion of reports of randomised trials published in languages other than English in systematic reviews. Health Technol Assess.

[55] Morrison A, Polisena J, Husereau D, Moulton K, Clark M, Fiander M (2012). The effect of English-language restriction on systematic review-based meta-analyses: a systematic review of empirical studies. Int J Technol Assess Health Care.

[56] Spaulding SE, Chen T, Chou LS (2012). Selection of an above or below-ankle orthosis for individuals with neuropathic partial foot amputation: a pilot study. Prosthet Orthot Int.

[57] Dillon MP, Fatone S, Hodge MC (2007). Biomechanics of ambulation after partial foot amputation: a systematic literature review. J Prosthet Orthot.

[58] Moher D, Liberate A, Tetzlaff J, Altman D (2009). Preferred Reporting Items for Systematic Reviews and Meta-Analyses: the PRISMA Statement. PLoS Med.

[59] Chen X (2010). The declining value of subscription-based abstracting and indexing services in the new knowledge dissemination era. Serials Rev.

[60] Falagas ME, Pitsouni EI, Malietzis GA, Pappas G (2008). Comparison of PubMed, Scopus, Web of Science, and Google Scholar: strengths and weaknesses. FASEB J.

[61] Löhönen J, Isohanni M, Nieminen P, Miettunen J (2010). Coverage of the bibliographic databases in mental health research. Nord J Psychiatry.

[62] Patla AE, Shumway-Cook A (1999). Dimensions of mobility: defining the complexity and difficulty associated with community mobility. J Aging Phys Act.

[63] Reed KL, Jelson SS (1992). Concepts of occupational therapy.

[64] Organization WH (2001). International Classification of Functioning, Disability and Health.

[65] Ozturk H, Dillon MP, Duke EJ, Kennedy-Jones MK (2014). Experience of sequential partial foot and transtibial amputation: a narrative enquiry.

[66] Schuch C, Pritham CH (1994). International forum—International Standards Organization terminology: application to prosthetics and orthotics. J Prosthet Orthot.

[67] Cooper H, Ribble RG (1989). Influences on the outcome of literature searches for integrative research reviews. Sci Comm.

[68] Law M, Stewart D, Pollock N, Bosch J, Westmorland M (1998). Critical Review Form—quantitative studies.

[69] Law M, Stewart D, Pollock N, Bosch J, Westmorland M (1998). Guidelines for Critical Review Form—quantitative studies.

[70] Law M, MacDermaid J (2008). Evidence-based rehabilitation: a guide to practice.

[71] Stern P (2005). A holistic approach to teaching evidence-based practice. Am J Occup Ther.

[72] Thomas B, Ciliska D, Dobbins M, Micucci S (2004). A process for systematically reviewing the literature: providing the research evidence for public health nursing interventions. Worldviews Evid Based Nurs.

[73] Cochrane Consumers and Communication Review Group C. Data extraction template. 2013. http://cccrg.cochrane.org/author-resources. Accessed 17.3.2015.

[74] Richardson A, Dillon MP. User experience of transtibial prosthetic liners: a systematic review. Prosth Orthot Int. In press. 10.1177/0309364616631343 26932981

[75] Buscemi N, Hartling L, Vandermeer B, Tjosvold L, Klassen TP (2006). Single data extraction generated more errors than double data extraction in systematic reviews. J Clin Epidemiol.

[76] Jones AP, Remmington T, Williamson PR, Ashby D, Smyth RL (2005). High prevalence by low impact of data extraction and reporting errors were found in Cochrane systematic reviews. J Clin Epidemiol.

[77] Sinha R, Van Den Heuvel W (2011). A systematic literature review of quality of life in lower limb amputees. Disabil Rehabil.

[78] Guyatt GH, Oxman AD, Kunz R, Vist GE, Falck-Ytter Y, Schunemann HJ (2008). What is ‘quality of evidence’ and why is it important to clinicians?. BMJ Case Reports.

