# Erratum to: Describe the outcomes of dysvascular partial foot amputation and how these compare to transtibial amputation: a systematic review protocol for the development of shared decision making resources

**DOI:** 10.1186/s13643-015-0171-7

**Published:** 2016-01-04

**Authors:** Michael P. Dillon, Stefania Fatone, Matthew Quigley

**Affiliations:** Discipline of Prosthetics and Orthotics, College of Science, Health and Engineering, La Trobe University, Melbourne, 3086 Australia; Northwestern University Prosthetic and Orthotic Centre, Feinberg School of Medicine, Northwestern University, 680 N Lake Shore Drive, Suite 1100, Chicago, 60611 IL USA

## Erratum

After publication of [1] it came to the publisher’s attention that the title of the article was displayed incorrectly. The correct title is “Describing the outcomes of dysvascular partial foot amputation and how these compare to transtibial amputation: a systematic review protocol for the development of shared decision making resources.”

## References

[1] Dillon PM, Fatone S, Quigley M (2015). Describe the outcomes of dysvascular partial foot amputation and how these compare to transtibial amputation: a systematic review protocol for the development of shared decision-making resources. Syst Rev.

