# Supplementary material for: Describe the outcomes of dysvascular partial foot amputation and how these compare to transtibial amputation: a systematic review protocol for the development of shared decision-making resources
Source: Syst Rev. 2015 Dec 4;4:173. doi: 10.1186/s13643-015-0161-9 (PMC4670495; doi:10.1186/s13643-015-0161-9)
Supplement: Additional file 2: — Grant award letter from the funding body. [file 13643_2015_161_MOESM2_ESM.pdf]

American Orthotic & Prosthetic Association

July 16, 2015

Michael Dillon, PhD
La Trobe University, Department of Rehabilitation, Nutrition and Sport
Victoria, Australia 3086

VIA Email: michael.dillon@latrobe.edu.au

Dear Dr. Dillon:

We are pleased to inform you that the Board of Directors of the American Orthotic and Prosthetic Association has authorized your systematic review project "Evaluating Outcomes of Dysvascular Partial Foot and Transtibial Amputation: A Systematic Review and Development of Shared Decision Making Resources" for funding of $59005. A review of your proposal in response to the RFP was also conducted by the Board of Directors for the Center for Orthotic and Prosthetic Learning which provided independent input and concurred with the recommendation of the AOPA Board of Directors.

AOPA has approved the funding for your project to be paid in two installments with the first half disbursed upon receipt of instructions as to the payee along with the appropriate SSN or Tax ID number for the advance, information which is required by the IRS, and the second installment upon receipt of the Interim Report, funds released on or about February 1, 2016. This grant is provided with the following stipulations; and in accepting this grant you agree to abide by and meet the timetable/deadlines set out below:

1. The funds are restricted to use for the specific project, investigator(s) and institution(s) stated in your grant proposal. Within that project, the funds are largely unrestricted and can be used for whatever phase of the research you deem necessary as noted in your proposed budget, subject however to the limitations of the Research Award's announcement.

2. Publications that arise from this effort should acknowledge that the work was supported by an AOPA Research Award.

3. You acknowledges that the end product was commissioned and supported by AOPA, but your institution shall have freedom to publish results without either prior approval or interference from AOPA. AOPA acknowledges that the end product was commissioned and supported by AOPA, but your institution(s) shall have freedom to publish results without either prior approval or interference from AOPA.

4. You acknowledge underlying responsibility to conduct the work in accordance with the specific proposal you submitted in response to the RFP.

Schedule/Deadlines and Key Deliverables

- Deliver a draft systematic review for review by the expert working group by 1st March, 2016.
- Submit manuscript peer-reviewed medical journal for publication By April 31, 2016.
- In collaboration with the expert working group, and based on the findings of the systematic review, draft shared-decision making resources (i.e., a discussion guide and a decision aid) for review in June 2016 with a final version due in October 2016.
- Presentation of the systematic literature review at the AOPA National Assembly September 8-11, 2016 in Boston, MA.

An interim report will be due about six months into the project or February 1, 2016 and a final report by October 1, 2016. Please provide instructions as to how the check should be prepared and to what address it should be mailed. Please sign as indicated below and return this together with materials referenced above, including the SSN or Tax ID number which AOPA will use to report the grant to the IRS and to initiate the process for release of AOPA's initial check.

Congratulations on your selection and we look forward to your contribution to a better understanding of this topic.

Very truly yours,

Thomas F. Fise, JD
Executive Director

Signed: Date: 17th July 2015
Principle Investigator
